# Molecular magnetic switch for a metallofullerene

**DOI:** 10.1038/ncomms7468

**Published:** 2015-03-03

**Authors:** Bo Wu, Taishan Wang, Yongqiang Feng, Zhuxia Zhang, Li Jiang, Chunru Wang

**Affiliations:** 1 Key Laboratory of Molecular Nanostructure and Nanotechnology, Beijing National Laboratory for Molecular Sciences, Institute of Chemistry, Chinese Academy of Sciences, Beijing 100190, China

## Abstract

The endohedral fullerenes lead to well-protected internal species by the fullerene cages, and even highly reactive radicals can be stabilized. However, the manipulation of the magnetic properties of these radicals from outside remains challenging. Here we report a system of a paramagnetic metallofullerene Sc_3_C_2_@C_80_ connected to a nitroxide radical, to achieve the remote control of the magnetic properties of the metallofullerene. The remote nitroxide group serves as a magnetic switch for the electronic spin resonance (ESR) signals of Sc_3_C_2_@C_80_ via spin–spin interactions. Briefly, the nitroxide radical group can ‘switch off’ the ESR signals of the Sc_3_C_2_@C_80_ moiety. Moreover, the strength of spin–spin interactions between Sc_3_C_2_@C_80_ and the nitroxide group can be manipulated by changing the distance between these two spin centres. In addition, the ESR signals of the Sc_3_C_2_@C_80_ moiety can be switched on at low temperatures through weakened spin–lattice interactions.

## Abstract (web summary)

Endohedral fullerenes are known to stabilize reactive radicals; however, the external magnetic manipulation of these species’ remains challenging. Here, the authors link a nitroxide radical to a paramagnetic fullerene system and are able to alter the spin behaviour of the fullerene via spin–spin interactions.

## Graphical abstract

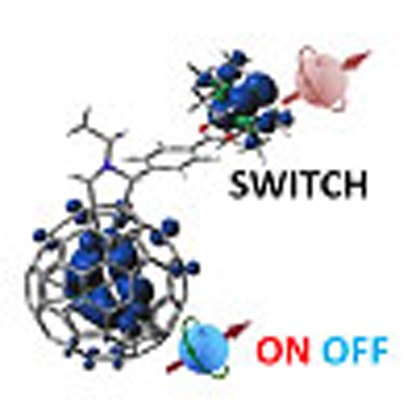

Endohedral fullerenes are constructed by putting atoms or clusters inside fullerene cages, which isolates the internal species with environments, so even those high-reactive species can be well stabilized inside the fullerene cages^1^,^2^,^3^,^4^,^5^,^6^. For example, paramagnetic endohedral fullerenes such as N@C_60_ (refs 7, 8, 9, 10), Sc@C_82_ (refs 11, 12), Y@C_82_ (refs 13, 14), Sc_3_C_2_@*I*_*h*_-C_80_ (refs 15, 16, 17) and so on, encapsulating radicals inside the fullerene cages, show also remarkable high stability, and they can be kept in air under room temperature for a long time, especially for endohedral metallofullerenes (EMFs). Considering the paramagnetic endohedral fullerenes usually show long electron spin relaxation and coherence times, they are expected to have potential applications in many fields such as spin labelling, spintronics, quantum computing and so on^18^.

For paramagnetic EMFs, the electronic spin resonance (ESR) technique is a powerful tool to detect the spin distributions and spin–nucleus couplings on internal species^19^,^20^,^21^. By means of ESR, it was revealed that the magnetic property of the internal species can be roughly manipulated by changing the dynamic movement of internal species. For example, under room temperature the internal Y_2_ cluster in Y_2_@C_79_N has a free rotation that leads to a symmetric ESR pattern, but along with the temperature decreasing, the free motion of Y_2_ is hindered, leading to spin anisotropy and an asymmetric ESR pattern^22^. In addition, the spin characters and couplings in Sc_3_C_2_@C_80_ was observed to change largely upon chemical modification of the fullerene cage due to the restricted Sc_3_C_2_ cluster^17^.

For better applying the paramagnetic EMFs in quantum information process and molecular devices, however, it is still a challenge to finely manipulate their magnetic property. Recently, Turro *et al*. chemically modified the H_2_@C_60_ with a nitroxide radical, and observed an indirect but strong magnetic communication between the electron spin of nitroxide paramagnet and the nuclear spin of encaged H_2_ (refs 23, 24, 25, 26, 27, 28). This finding provides a valuable clue for us on manipulating the magnetic property of paramagnetic EMFs via a foreign paramagnet^29^. Since a strong spin–spin interaction between the paramagnetic fullerene molecule and the paramagnet is expected, thus the magnetic property of paramagnetic EMFs may be controlled by the attached paramagnet.

Herein, we report detailed studies on the fine manipulation for paramagnetic Sc_3_C_2_@C_80_ by connecting it with a paramagnet of nitroxide radical. The target system FSc_3_C_2_@C_80_PNO^·^ contains two kinds of spins localizing on Sc_3_C_2_@C_80_ and nitroxide radical, respectively. The remote nitroxide group serves as a magnetic switch for the ESR signals of Sc_3_C_2_@C_80_ through spin–spin interactions. The paramagnetic properties of the metallofullerene Sc_3_C_2_@C_80_ can be delicately adjusted by changing the temperature, varying the distance between the two spin centres, or simply quenching the nitroxide radical.

## Results

### Preparation of metallofullerene and its derivatives

**Figure 1:**
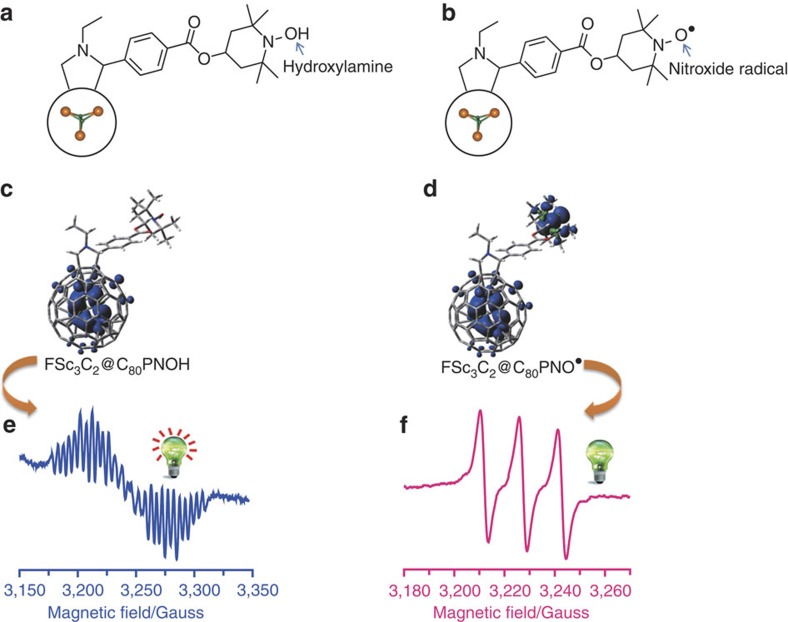
Magnetic switch for the ESR signals of Sc_3_C_2_@C_80_. (**a**) The structure of FSc_3_C_2_@C_80_PNOH. (**b**) The structure of FSc_3_C_2_@C_80_PNO^·^. (**c**) The calculated structure and spin density distributions of FSc_3_C_2_@C_80_PNOH. (**d**) The calculated structure and spin density distributions of FSc_3_C_2_@C_80_PNO^·^. (**e**) The ESR spectrum of FSc_3_C_2_@C_80_PNOH at 293 K in toluene. (**f**) The ESR spectrum of FSc_3_C_2_@C_80_PNO^·^ at 293 K in toluene. The lamps in **e** and **f** show the ‘on’ and ‘off’ states of Sc_3_C_2_@C_80_ ESR signals, respectively.

Metallofullerene Sc_3_C_2_@C_80_ was synthesized by the Kräschmer–Huffman arc-discharging method^30^ and isolated by multi-stage high-performance liquid chromatography. Two Sc_3_C_2_@C_80_ derivatives, FSc_3_C_2_@C_80_PNOH and FSc_3_C_2_@C_80_PNO^·^, were first synthesized through a Prato reaction^31^, respectively, as shown in Fig. 1a,b. The structures and spin density distributions of FSc_3_C_2_@C_80_PNOH and FSc_3_C_2_@C_80_PNO^·^ were calculated as well, as shown in Fig. 1c,d. The FSc_3_C_2_@C_80_PNOH has one spin centre that is localized on Sc_3_C_2_@C_80_, whereas the FSc_3_C_2_@C_80_PNO^·^ has two unpaired spins localizing on the Sc_3_C_2_@C_80_ moiety and nitroxide radical, respectively.

### The ESR analysis of metallofullerene with a nitroxide radical

Sc_3_C_2_@C_80_ is a typical paramagnetic endohedral fullerene. As reported previously, the ESR spectrum of the pristine Sc_3_C_2_@C_80_ shows a symmetric pattern with 21 resonant lines, however, those of Sc_3_C_2_@C_80_ derivatives showed a distorted pattern with a greatly increased amount of resonant lines^17^. Therefore, ESR spectroscopy was first employed to reveal the electron spin characters of FSc_3_C_2_@C_80_PNOH and FSc_3_C_2_@C_80_PNO^·^.

As shown in Fig. 1e, the ESR spectrum of FSc_3_C_2_@C_80_PNOH was measured and analysed. Because the *I*_*h*_ symmetry of Sc_3_C_2_@C_80_ is broken down after chemical modification, the original three equivalent scandium nuclei (*I*_Sc_=7/2) are classified into two groups in FSc_3_C_2_@C_80_PNOH, in which one group contains a single Sc nucleus (*g*=1.9948, hyperfine coupling constants (hfcc)=8.5 G), and the other group contains two equivalent Sc nuclei (hfcc=5.0 G). In comparison, the previously studied Sc_3_C_2_@C_80_ fulleropyrrolidine shows a similar ESR pattern with hfcc of 8.6 G (one Sc nucleus) and 4.8 G (two Sc nuclei), respectively^17^. These ESR results reveal that the FSc_3_C_2_@C_80_PNOH has a same reaction site with that of Sc_3_C_2_@C_80_ fulleropyrrolidine^17^.

However, the ESR study of FSc_3_C_2_@C_80_PNO^·^ showed only three resonant lines (*g*=2.0026, *a*=15.5 G) that are derived from nitroxide radical (*I*_N_=1), and the ESR signals of Sc_3_C_2_@C_80_ moiety were not observed, as illustrated in Fig. 1f. The current results reveal that the ESR signals of Sc_3_C_2_@C_80_ can be switched off by paramagnetic nitroxide radical through spin–spin interaction. Thus it is interesting that the nitroxide radical group can serve as a remote controller for the ESR signals of Sc_3_C_2_@C_80_ moiety. Vividly, if the ESR signals of Sc_3_C_2_@C_80_ moiety are regarded as an indicating lamp, the nitroxide radical group can switch it off.

To reveal the mechanism of how the ESR signals of Sc_3_C_2_@C_80_ moiety are switched off by the nitroxide radical, we synthesized FSc_3_N@C_80_PNO^·^ in a same way for comparison. Sc_3_N@C_80_ is a diamagnetic molecule, and no spin–spin interaction is expected for FSc_3_N@C_80_PNO^·^. The signal intensity of FSc_3_N@C_80_PNO^·^ was observed to be stronger than that of FSc_3_C_2_@C_80_PNO^·^ at the same concentration (0.151 p.p.m.). That is to say, the spin–spin interactions weaken both of the ESR signals of Sc_3_C_2_@C_80_ and nitroxide radical (Supplementary Fig. 1).

For FSc_3_C_2_@C_80_PNO^·^, the spin–spin interactions can be expressed as below:


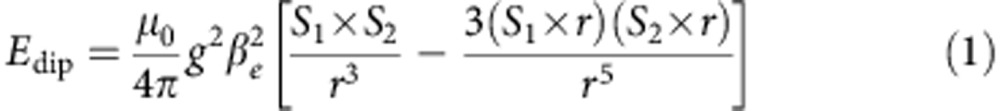


Where *E*_dip_ is the energy of dipolar coupling, and *r* is the dipole–dipole distance.

In fact, the spin–spin interactions between Sc_3_C_2_@C_80_ and nitroxide radical broaden the resonance lines and lower the line intensity in the meantime, so the line width (Δ*H*) is adopted to represent the ESR line intensity and interpret the spin–spin interactions^32^,^33^.

In general, for paramagnetic molecules the ESR line width is inversely proportional to the relaxation time (*T*), including the spin–lattice relaxation time (*T*_1_) and the spin–spin relaxation time (*T*_2_):


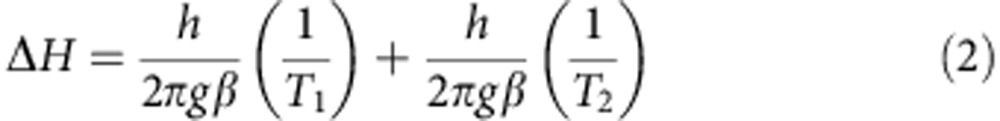


Therefore, the strong dipole–dipole interactions between nitroxide radical and Sc_3_C_2_@C_80_ in FSc_3_C_2_@C_80_PNO^·^ reduced the spin–spin relaxation time (*T*_2_), resulting in decreased ESR signals of both Sc_3_C_2_@C_80_ and nitroxide radical moieties.

Note that the transformation between FSc_3_C_2_@C_80_PNO^·^ and FSc_3_C_2_@C_80_PNOH is reversible, that is, the nitroxide radical in FSc_3_C_2_@C_80_PNO^·^ turns into the corresponding hydroxylamine derivative (FSc_3_C_2_@C_80_PNOH) using *p*-toluenesulfonohydrazide, and the FSc_3_C_2_@C_80_NOH can be back to FSc_3_C_2_@C_80_PNO^·^ by means of oxidation with copper acetate (Supplementary Fig. 2). Therefore, the magnetic property of Sc_3_C_2_@C_80_ can be easily manipulated by a chemical method, and the remote nitroxide group serves as a switch in this process.

### The distance-dependent ESR signals

**Figure 2:**
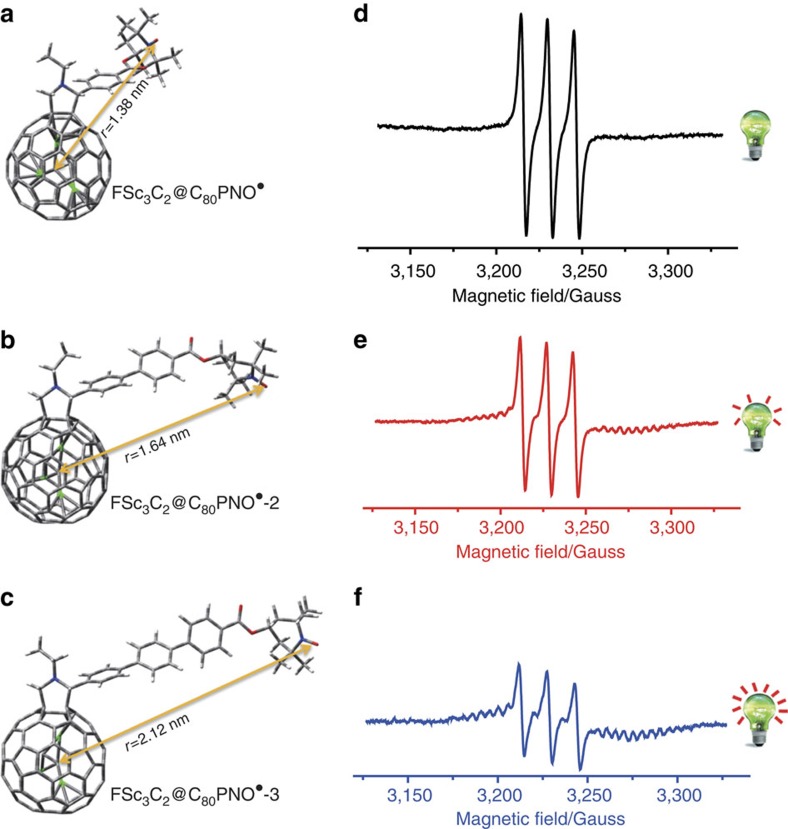
Distance-dependent ESR signals of Sc_3_C_2_@C_80_ derivatives. (**a**–**c**) Structures of FSc_3_C_2_@C_80_PNO^·^, FSc_3_C_2_@C_80_PNO^·^-2 and FSc_3_C_2_@C_80_PNO^·^-3. (**d**–**f**) ESR signals of FSc_3_C_2_@C_80_PNO^·^, FSc_3_C_2_@C_80_PNO^·^-2 and FSc_3_C_2_@C_80_PNO^·^-3 at 293 K. The lamps in **d**–**f** show the strengthened ESR signals of Sc_3_C_2_@C_80_ moiety along with the enlarged distance from nitroxide radical.

On the basis of equation (1), the spin–spin interactions between Sc_3_C_2_@C_80_ and nitroxide radical moieties can be efficiently reduced by elongating their distance, thus two other Sc_3_C_2_@C_80_ and nitroxide radical derivatives, that is, FSc_3_C_2_@C_80_PNO^·^-2 and FSc_3_C_2_@C_80_PNO^·^-3, were synthesized with longer distances between these two spin centres. The pulsed ESR measurements on FSc_3_C_2_@C_80_PNO^·^ and FSc_3_C_2_@C_80_PNO^·^-2 revealed that the *T*_2_ of nitroxide radical becomes longer when the distance of these two spins increases (Supplementary Fig. 3). As shown in Fig. 2, it is obvious that the ESR signals of Sc_3_C_2_@C_80_ also gradually boost up along with the distance increasing, and this process is like lighting a lamp and making it brighter.

However, the increased chain length between Sc_3_C_2_@C_80_ and nitroxide radical would result in a strengthened spin–lattice interaction, which will bring another line-broadening effect for both nitroxide radical and Sc_3_C_2_@C_80_ moiety. The spin–lattice relaxation time (*T*_1_) can be expressed as below:


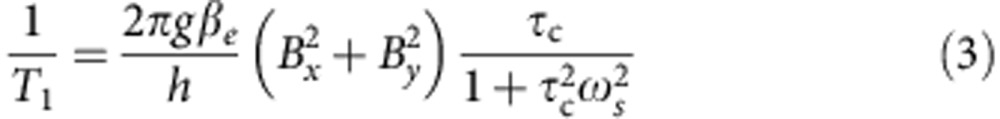


Where 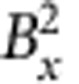 and 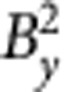 are mean square amplitudes of the fluctuating fields along the *x*- and *y*-directions, and *τ*_c_ is the correlation time of the motion that causes the fluctuation. For molecules with a spherical shape, *τ*_c_ in liquid solution corresponds to the rotational correlation time *τ*_r_, which can be approximated by the Stokes–Einstein relation:


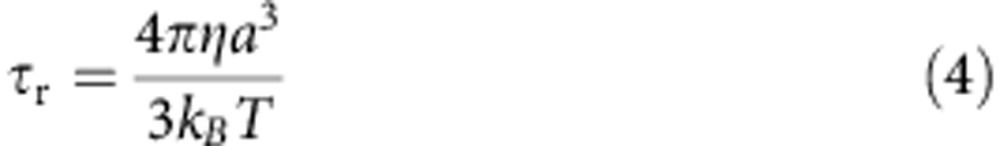


Where *a* is the rotationally effective radius of the molecule, and *η* is the viscosity of the solvent. In liquids with a low viscosity (*τ*^*2*^_c_*ω*^*2*^_*s*_≈1), the *T*_1_ is dependent of *a*^*3*^, and *T*_1_ decreases with increasing the rotationally effective radius of the molecule. Therefore, increasement of the molecular size would shorten the *T*_1_, leading to weaker ESR signals.

Since FSc_3_C_2_@C_80_PNO^·^, FSc_3_C_2_@C_80_PNO^·^-2 and FSc_3_C_2_@C_80_PNO^·^-3 are all rigid structural molecules, the intramolecular dipolar coupling strength (*D*) can be estimated, in which the coupling strength of FSc_3_C_2_@C_80_PNO^·^ with *r*=1.38 nm was estimated to be about 24.3 MHz following the classical point dipole approximation, and those of FSc_3_C_2_@C_80_PNO^·^-2 and FSc_3_C_2_@C_80_PNO^·^-3 were estimated to be about 11.2 and 5.48 MHz, respectively.

### The temperature-dependent ESR signals

**Figure 3:**
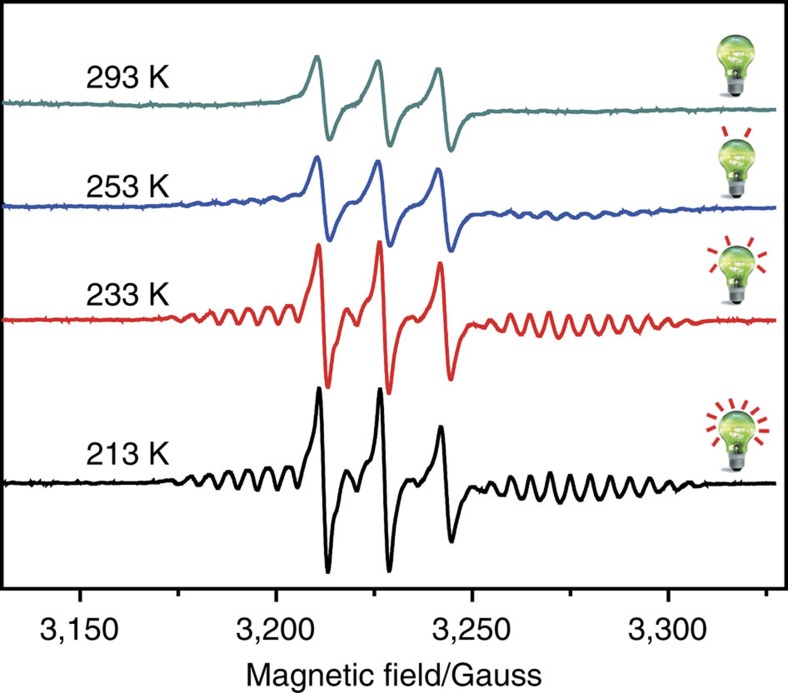
Temperature-dependent ESR signals of Sc_3_C_2_@C_80_ derivatives. The ESR spectra of FSc_3_C_2_@C_80_PNO^·^ at variable temperatures in toluene solution. The lamps represent the strengthened ESR signals of Sc_3_C_2_@C_80_ moiety along with the decreased temperatures.

It is known that the spin–lattice relaxation time (*T*_1_) of unpaired spin is tightly related to the temperature. As expressed in equation (3), the decreasement of temperature would reduce the *B*_*x*_ and *B*_*y*_, and then increase the *T*_1_ and lead to higher ESR line intensity. Therefore, the temperature-dependent ESR studies of FSc_3_C_2_@C_80_PNO^·^ were performed, as shown in Fig. 3. It can be observed that no ESR signal of Sc_3_C_2_@C_80_ was observed at 293 K, but since 253 K, the ESR signals of Sc_3_C_2_@C_80_ appeared together with three strong resonant lines of nitroxide radical, and continuously increased along with the temperature further decreasing. Finally at 213 K, the ESR signals of Sc_3_C_2_@C_80_ moiety can be clearly observed. The electrostatic spin–phonon interaction was also analysed for these temperature-dependent ESR spectra^34^. As the lowest temperature in our system is 213 K, under this condition the FSc_3_C_2_@C_80_PNO^·^ toluene solution is still in liquid state, so the spin–phonon interaction is rather small and negligible. From the temperature-dependent ESR spectra, it can be seen that the temperature also can light the signals of Sc_3_C_2_@C_80_ moiety in FSc_3_C_2_@C_80_PNO^·^ and make it brighter.

Moreover, it should be noted that the ESR signals of the nitroxide were also enhanced. Therefore, along with the temperature decreasing, the prolonged *T*_1_ enhances not only the ESR signals of Sc_3_C_2_@C_80_ moiety, but also those of the nitroxide radical (Supplementary Fig. 4). The line widths of Sc_3_C_2_@C_80_ moiety and nitroxide radical are listed in Table 1.

## Discussion

Through connecting the paramagnetic metallofullerene Sc_3_C_2_@C_80_ with a nitroxide radical, we have realized the manipulation of ESR signals of Sc_3_C_2_@C_80_. The remote nitroxide group serves as a magnetic switch for ESR signals of Sc_3_C_2_@C_80_, that is, the paramagnetic nitroxide group can ‘switch off’ the ESR signals of Sc_3_C_2_@C_80_ moiety. It was revealed that the spin–spin interactions between Sc_3_C_2_@C_80_ and nitroxide radical play a key role in realizing this kind of magnetic switch. Moreover, through increasing the distance between Sc_3_C_2_@C_80_ and nitroxide radical, or decreasing the temperature, we can finely adjust the paramagnetic property of Sc_3_C_2_@C_80_.

Such controllable paramagnetism and switchable ESR signals have potential applications in quantum information processing and molecular devices. For example, these magnetic molecules can be fabricated to a single molecule membrane for data storage considering their transferrable two electron spin states (0/1), which can be written and read out by means of scanning tunnelling microscope. In addition, these magnetic molecules can be utilized as a probe for the reaction transition state considering the susceptible magnetic switch of the nitroxide group.

## Methods

### Synthesis of Sc_3_C_2_@C_80_ and Sc_3_N@C_80_ derivatives

Sc_3_C_2_@C_80_ and Sc_3_N@C_80_ were heated with *N*-ethylglycine and 2,2,6,6 tetramethylpiperidine-1-oxyl 4-formylbenzoate (1a) (Supplementary Fig. 5), which were synthesized as literature methods^24^ at 120 °C to give corresponding fulleropyrrolidines with yields of nearly 50% in toluene solution for 15 and 50 min, respectively. Pure FSc_3_C_2_@C_80_PNO^·^ and FSc_3_N@C_80_PNO^·^ were isolated by HPLC using Buckyprep column (Supplementary Fig. 6). FSc_3_C_2_@C_80_PNO^·^-2 and FSc_3_C_2_@C_80_PNO^·^-3 were synthesized according to same procedure used for compound FSc_3_C_2_@C_80_PNO^·^, except 2,2,6,6 tetramethylpiperidine-1-oxyl 4'-formylbiphenyl-4-carboxylate (2a) and 2,2,6,6 tetramethylpiperidine-1-oxyl 4'-p-terphenyl-4-carboxylate (3a) (Supplementary Fig. 7) were used.

### Synthesis of FSc_3_C_2_@C_80_PNOH

To a solution of ~0.5 mg of the nitroxide derivative FSc_3_C_2_@C_80_PNO^·^ in ~2 ml toluene was added ~1 mg *p*-toluenesulfonohydrazide, and stirred under air for about 15 min.

### Characterization of metallofullerene derivatives

Ultraviolet/visible–near-infrared spectra of purified metallofullerene derivatives (Supplementary Fig. 8) were collected on Lambda 950 UV/Vis/NIR Spectrometer (PerkinElmer Instruments). ^1^H NMR spectra of FSc_3_N@C_80_PNOH was measured in chloroform-*d* on a Bruker 600 MHz spectrometer (Supplementary Fig. 9).

### ESR measurements of metallofullerene derivatives

ESR spectra were measured on a JEOL JEF FA200 X-band spectrometer (Supplementary Fig. 10). The samples were degassed and the oxygen was removed from the solutions. All of the samples are dissolved in toluene solution at the same concentration.

### Calculations on Sc_3_C_2_@C_80_ derivatives

Density functional theory calculations were investigated by Perdew, Burke and Enzerhof/double numerical plus polarization using the DMol3 code in Accelrys Materials Studio^35^,^36^.

## Author contributions

T.W. conceived and designed the experiments. T.W. and C.W. wrote the paper. Experiments were carried out by B.W. The ESR data were analysed by T.W. and B.W. Calculations were carried out by Z.Z. All authors discussed the results and contributed to manuscript preparation.

## Additional information

**How to cite this article:** Wu, B. *et al*. Molecular magnetic switch for a metallofullerene. *Nat. Commun.* 6:6468 doi: 10.1038/ncomms7468 (2015).

## Supplementary Material

Supplementary Information Supplementary Figures 1-10

## Figures and Tables

**Table 1:** ESR data.

Temperature (K)	Line width of nitroxide (G)	Line width of Sc_3_C_2_@C_80_ (G)
293	3.17	—
253	3.08	2.63
233	2.47	2.37
213	2.35	2.13

ESR, electronic spin resonance.

The ESR spectra line width of nitroxide and Sc_3_C_2_@C_80_ in FSc_3_C_2_@C_80_PNO^·^ at variable temperatures.
