# Supplementary material for: Molecular magnetic switch for a metallofullerene
Source: Nat Commun. 2015 Mar 3;6:6468. doi: 10.1038/ncomms7468 (PMC4366484; doi:10.1038/ncomms7468)
Supplement: Supplementary Information — Supplementary Figures 1-10 [file ncomms7468-s1.pdf]

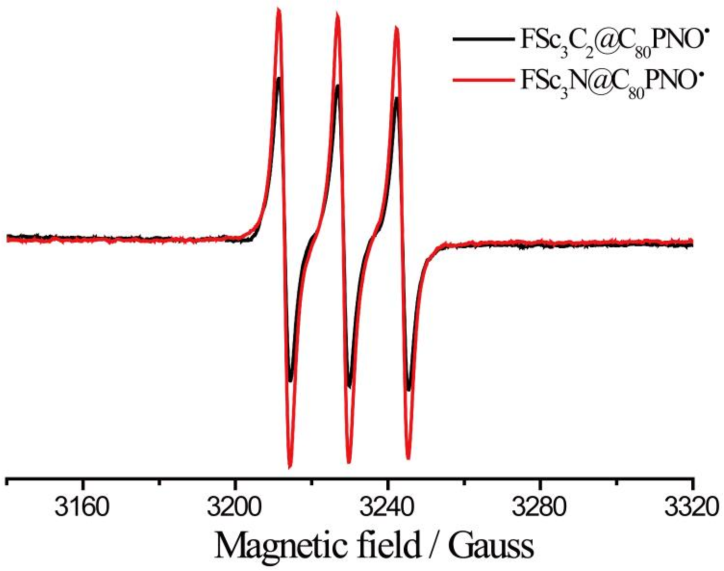

Supplementary Figure 1. The ESR spectra of $FSc_3N@C_{80}PNO^{\bullet}$ and $FSc_3C_2@C_{80}PNO^{\bullet}$. The ESR spectra of $FSc_3N@C_{80}PNO^{\bullet}$ and $FSc_3C_2@C_{80}PNO^{\bullet}$ at the same concentration in toluene solution at room temperature (ΔH of $FSc_3N@C_{80}PNO^{\bullet}$ is 3.01 G and ΔH of $FSc_3C_2@C_{80}PNO^{\bullet}$ is 3.36 G).

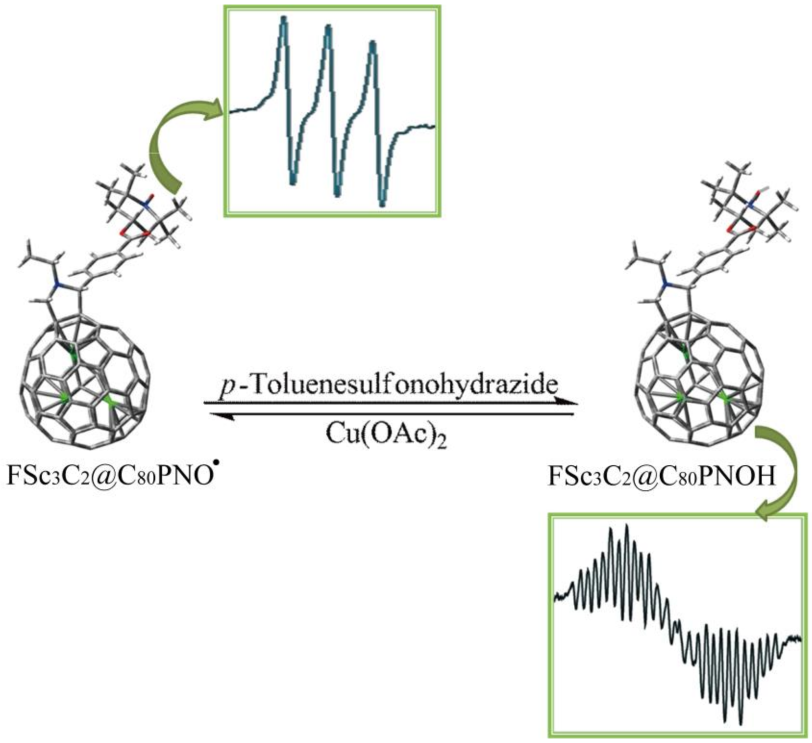

Supplementary Figure 2. The transformation and ESR spectra of Sc_3C_2@C_{80} derivatives. The nitroxide radical in FSc_3C_2@$C_{80}PNO^{\bullet}$ turns into the corresponding hydroxylamine derivative (FSc_3C_2@$C_{80}PNOH$) using *p*-Toluenesulfonohydrazide, and the FSc_3C_2@$C_{80}PNOH$ is back to FSc_3C_2@$C_{80}PNO^{\bullet}$ by means of oxidation with copper acetate at room temperature in toluene solution.

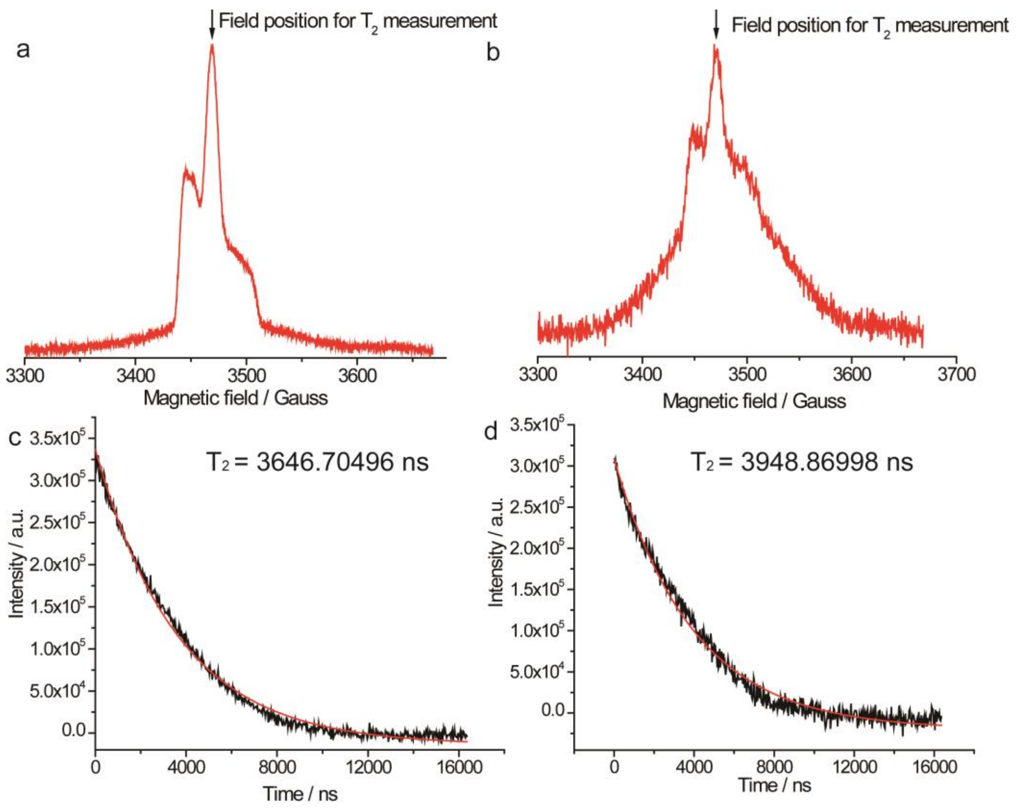

Supplementary Figure 3. The pulsed ESR measurements of $Sc_3C_2@C_{80}$ derivatives. The Echo detected field sweep and T_2 measurement are performed at 20K using 2 pulse Hann echo sequence. The pulse length is pi/2: 20 ns. The Echo detected field sweep for **a**, $FSc_3C_2@C_{80}PNO^{\bullet}$ and **b**, $FSc_3C_2@C_{80}PNO^{\bullet}$-2 are at the same experimental conditions. Echo decay curves for **c**, $FSc_3C_2@C_{80}PNO^{\bullet}$ and **d**, $FSc_3C_2@C_{80}PNO^{\bullet}$-2 are graphical depiction of the Hahn echo pulse sequence. The T_2 detection positions are marked with arrows. The red lines in **c** and **d** represent stretched exponential functions fit to the data.

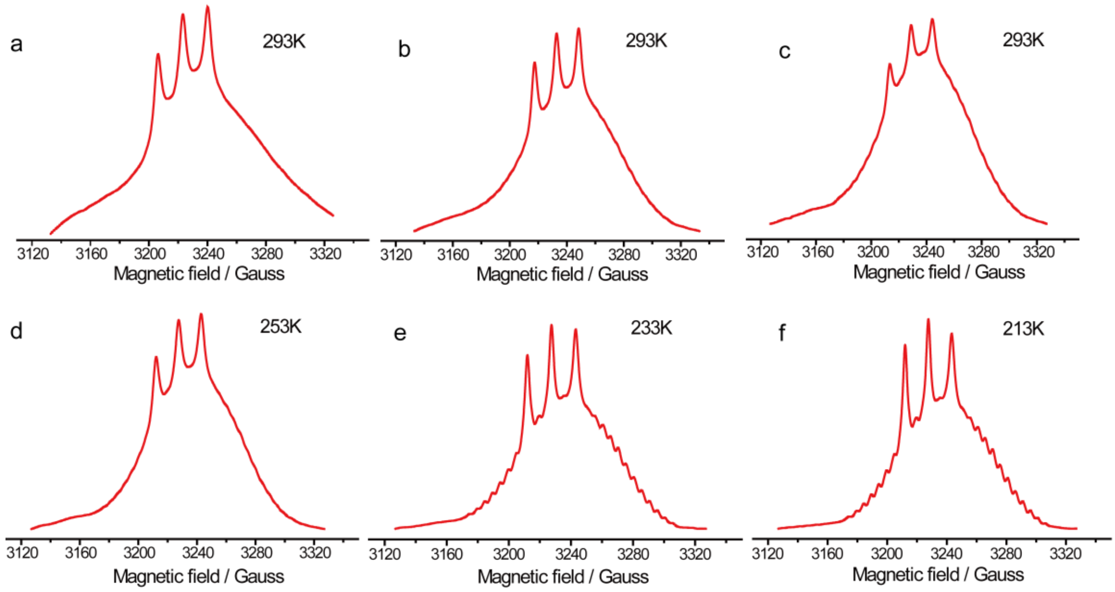

Supplementary Figure 4. The integral spectra of $Sc_3C_2@C_{80}$ derivatives. The integral spectra of three derivatives **a**, $FSc_3C_2@C_{80}PNO^{\bullet}$, **b**, $FSc_3C_2@C_{80}PNO^{\bullet}$-2 and **c**, $FSc_3C_2@C_{80}PNO^{\bullet}$-3 at 293K have been done, and the temperature-dependent integral ESR spectra of $FSc_3C_2@C_{80}PNO^{\bullet}$ at **d**, 253K, **e**, 233K and **f**, 213K were performed in toluene solution.

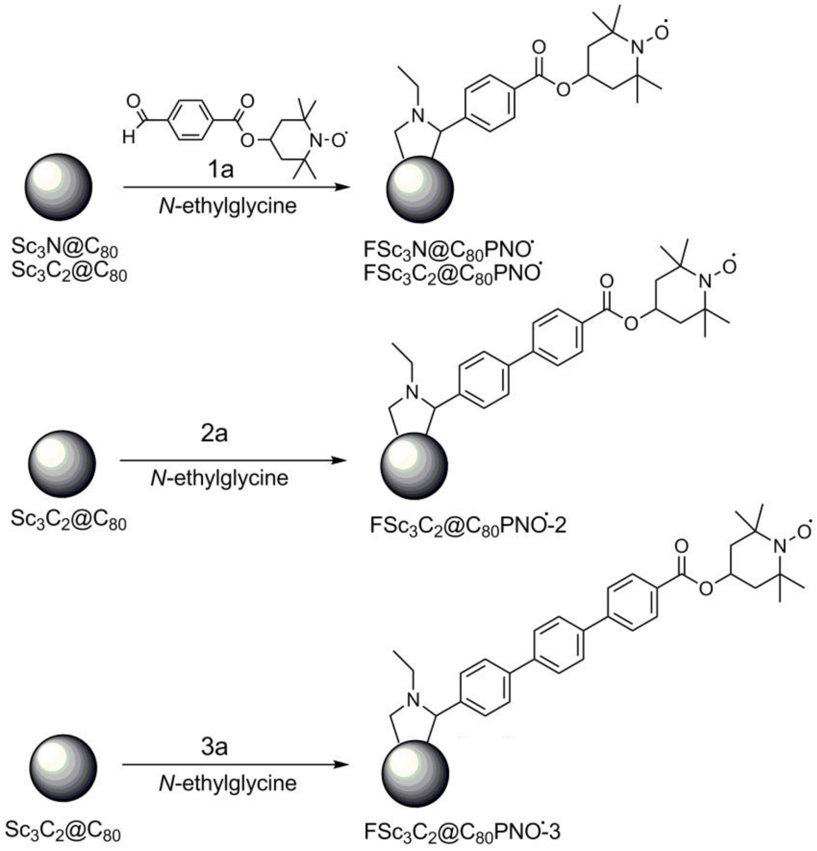

Supplementary Figure 5. The synthesis of $Sc_3N@C_{80}$ and $Sc_3C_2@C_{80}$ derivatives. The synthesis procedure of $FSc_3N@C_{80}PNO^•$, $FSc_3C_2@C_{80}PNO^•$, $FSc_3C_2@C_{80}PNO^•$-2 and $FSc_3C_2@C_{80}PNO^•$-3.

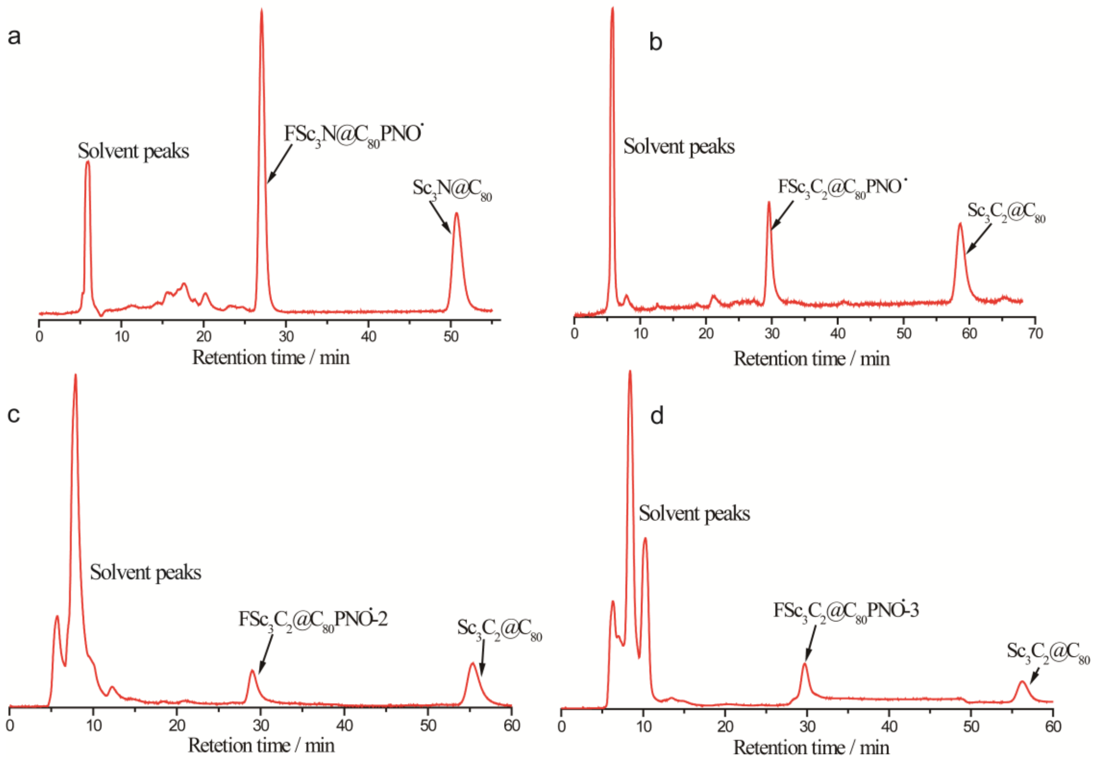

Supplementary Figure 6. The HPLC profiles of $Sc_3N@C_{80}$ and $Sc_3C_2@C_{80}$ derivatives. HPLC profiles of the cycloaddition of **a**, $FSc_3N@C_{80}PNO^{\bullet}$, **b**, $FSc_3C_2@C_{80}PNO^{\bullet}$, **c**, $FSc_3C_2@C_{80}PNO^{\bullet}$-2 and **d**, $FSc_3C_2@C_{80}PNO^{\bullet}$-3. HPLC conditions: 10×250 mm Buckyprep column, 12 mL/min flow rate with toluene, 330 nm detection.

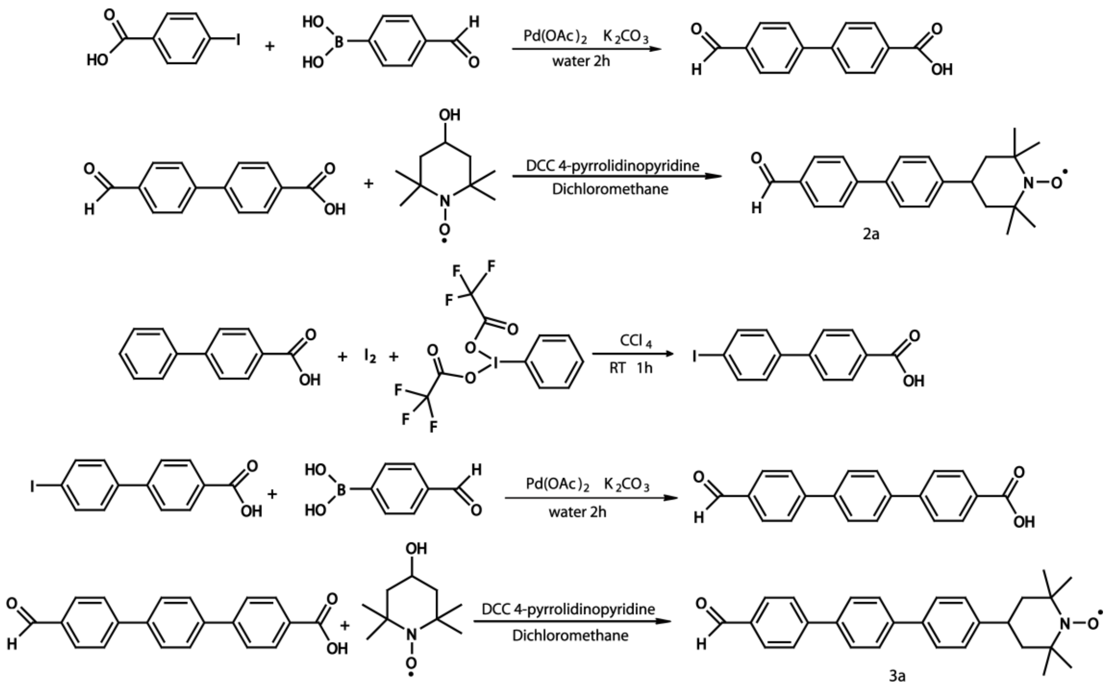

Supplementary Figure 7. The synthesis clues of nitroxide radical groups. The structures of 2,2,6,6 tetramethylpiperidine-1-oxyl 4'-formylbiphenyl-4-carboxylate (2a) and 2,2,6,6 tetramethylpiperidine-1-oxyl 4'-p-terphenyl-4-carboxylate (3a).

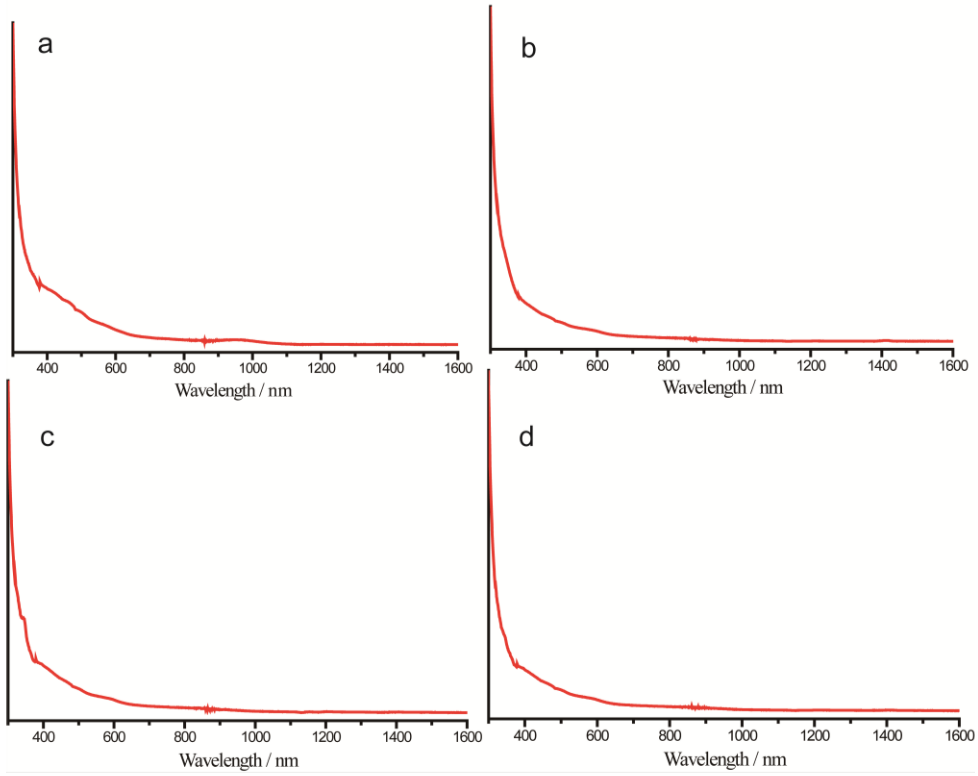

Supplementary Figure 8. The UV/Vis-NIR spectra of $Sc_3N@C_{80}$ and $Sc_3C_2@C_{80}$ derivatives. UV/Vis-NIR spectra of purified **a**, $FSc_3N@C_{80}PNO^•$, **b,** $FSc_3C_2@C_{80}PNO^•$, **c**, $FSc_3C_2@C_{80}PNO^•$-2 and **d**, $FSc_3C_2@C_{80}PNO^•$-3 in toluene.

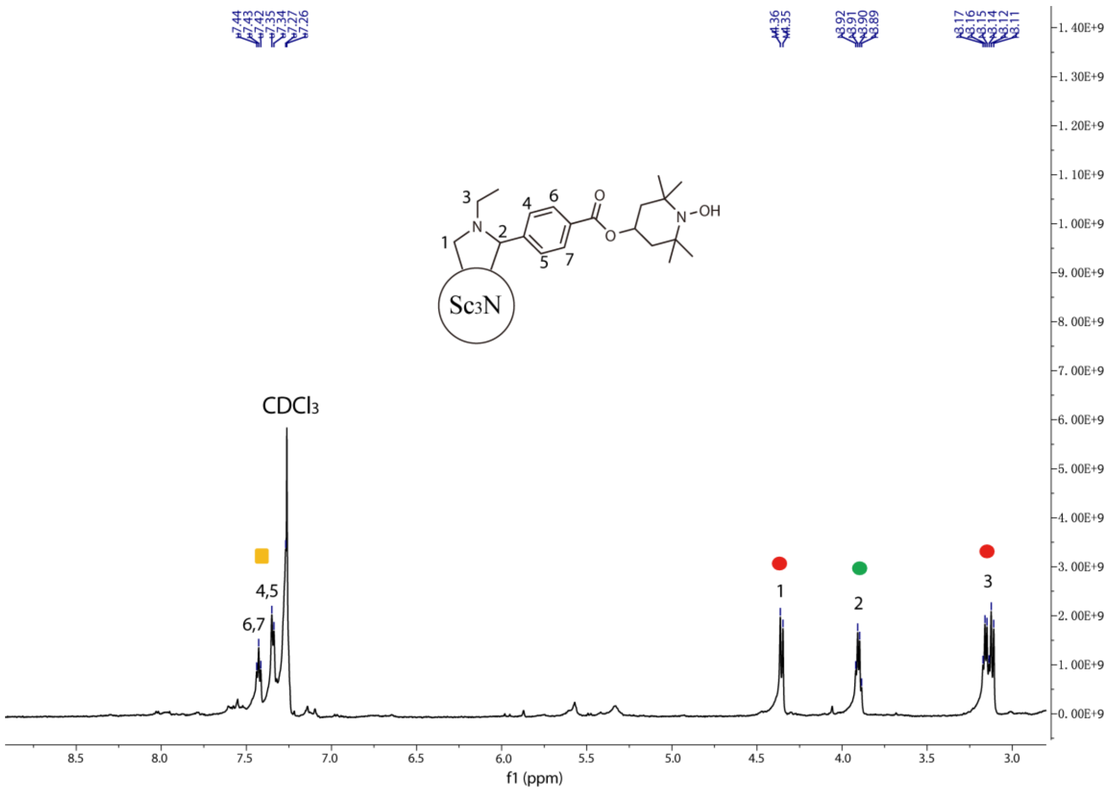

Supplementary Figure 9. ^{1}H NMR spectrum of FSc$_3$N@C$_{80}$PNOH. The ^{1}H NMR spectrum of FSc$_3$N@C$_{80}$PNOH between 3 and 9 ppm at 600 MHz in chloroform-*d* (CDCl$_3$) at 293 K. Signals marked by red and green circles are attributed respectively to the pyrrolidine geminal and methyne protons. The yellow square stands for aromatic protons.

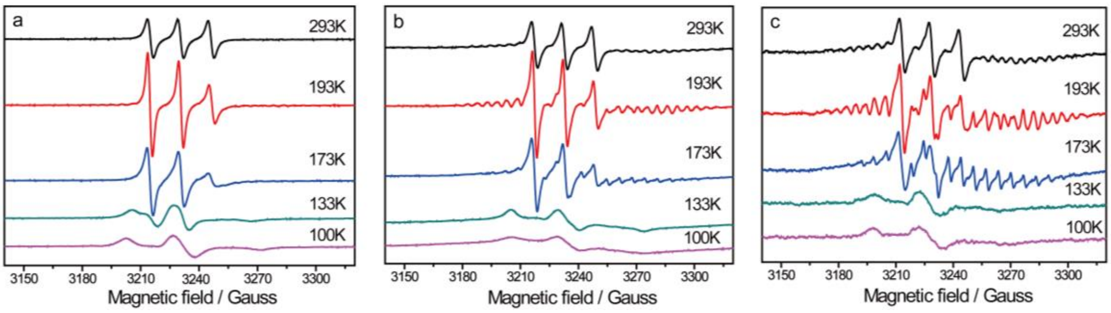

Supplementary Figure 10. The ESR spectra of $Sc_3C_2@C_{80}$ derivatives. The ESR spectra of three derivatives **a**, $FSc_3C_2@C_{80}PNO^{\bullet}$, **b**, $FSc_3C_2@C_{80}PNO^{\bullet}$-2 and **c**, $FSc_3C_2@C_{80}PNO^{\bullet}$-3 at low temperature in toluene solution.
